# Cytochrome P450arom, androgen and estrogen receptors in pig sperm

**DOI:** 10.1186/1477-7827-5-23

**Published:** 2007-06-06

**Authors:** Vittoria Rago, Saveria Aquila, Rocco Panza, Amalia Carpino

**Affiliations:** 1 Department of Cell Biology, Faculty of Pharmacy, University of Calabria, 87030 Arcavacata di Rende, Cosenza, Italy; 2 Department of Pharmaco-Biology, Faculty of Pharmacy, University of Calabria, 87030 Arcavacata di Rende, Cosenza, Italy; 3 Swine Artificial Insemination Centre, APA, Cosenza, Italy

## Abstract

**Background:**

Androgens and estrogens are crucial for mammalian sperm differentiation but their role in biology of mature male gamete is not still defined. The expression of proteins involved in the biosynthesis and action of these steroid hormones has been demonstrated in human spermatozoa, but very few data have been reported in mature sperm from non human species. The purpose of the current study was to investigate the expression of aromatase (P450arom), estrogen (ERalpha/ERbeta) and androgen (AR) receptors in ejaculated spermatozoa of pig.

**Methods:**

The immunfluorescence experiments were carried out treating pig sperm with anti-P450arom, anti-ERalpha, anti-ERbeta and anti-AR as primary antibodies, while Texas-Red/FITC conjugated IgG were applied as secondary antibodies. Furthermore, Western blot analysis was performed on sperm lysates.

**Results:**

Aromatase was immunolocalized in the sperm tail, ERalpha and AR were localised in the sperm midpiece, while ERbeta was confined in the acrosomal region of the male gamete. Immunoblots detected a ~52 kDa aromatase band, a ~110 kDa AR band, a ~67 kDa ERalpha and two ERbeta bands, at ~50 kDa and ~59 kDa.

**Conclusion:**

This is the first report demonstrating that pig ejaculated spermatozoa express aromatase, estrogen and androgen receptors with a differential intra-cellular localization revealing a specie-specific expression pattern. Therefore, pig sperm could be considered as a potential estrogen source while the different hormone cellular sites suggest distinct roles of androgens and estrogens in pig sperm physiology.

## Background

It is well known the role of androgen signaling in the regulation of mammalian spermatogenesis. In seminiferous tubules of rodents, primates and humans, androgen action is mediated by androgen receptors (AR) localised in peritubular myoid cells, in Sertoli cells and in spermatids, ensuring normal progression of germ cell maturational steps [1-3].

However, numerous studies have also suggested a key role of estrogens in male germ cell differentiation. In fact, proteins involved in estrogen biosynthesis and action, as aromatase (P450arom) and/or estrogen receptors (ERs), have been detected in developing sperm of numerous species such as rat [4-6], bank vole [7], rooster [8], boar [9,10], equine [11], brown bear [12], dog and cat [13], human [14,15].

There is only one known mammalian androgen receptor (AR) which is encoded by a gene located on the X chromosome [16] while there are two specific estrogen receptors, ERα and ERβ, each encoded by a unique gene, differing in the C-terminal ligand-binding domain and in the N-terminal *trans*-activation domain [17]. Several ER variant isoforms have been also recently identified, but their biological significance is still not defined [18].

Recent studies have been focused on androgen and estrogen involvement in mature sperm physiology. Aromatase, ERα, ERβ and AR have been detected in human sperm raising the hypothesis that estrogens and androgens could also regulate sperm functional properties [19-21].

Conversely, there are a few information regarding mature sperm from non human species; in fact, the only reported finding was the ERα expression in rat spermatozoa, suggesting a role of this estrogen mediator in the motility acquisition [22].

Despite the extensive studies on molecular mechanisms linked to porcine sperm fertility, the relation between sex hormones and mature sperm is, to date, scarcely known. Therefore, to contribute to a better understanding of pig sperm biology, this study has evaluated the expression of aromatase (P450arom), estrogen (ERα and ERβ) and androgen (AR) receptors in ejaculated spermatozoa from *Sus scrofa domestica*.

## Methods

### Animals and semen samples

The investigation has been conducted on semen from 7 fertile male pigs (*Sus scrofa domestica, Large White) *kept at " Swine Artificial Insemination Centre " (Rende, Cs, Italy). The animals were 24 to 30 month- old and their weights were from 280 to 320 kg. Individual fresh ejaculates were collected by the gloved hand method and filtered immediately by Universal Semen bags (Minitub, Tiefenbech, Germany). Semen was transported within half an hour to the laboratory, it was diluted 1:10 with TBS buffer (0.05 M TRIS-HCl, 0.15 M NaCl, pH 7.6) and centrifuged on a discontinuous Percoll density gradient (72%/90%) to remove bacteria and debris [23].

### Antibodies

Anti-AR primary antibody was mouse monoclonal 441(Santa Cruz Biotechnology, Ca, USA) which recognizes an epitope mapping at the C-terminus region of the human native AR. Anti-P450arom primary antibody was mouse monoclonal MCA 2077 (Serotec, Oxford, UK) which recognizes an epitope mapping at C- terminus region of the human placental aromatase. Anti-ERα primary antibody was mouse monoclonal F-10 (Santa Cruz Biotechnology, Ca, USA) which recognizes an epitope mapping at the C-terminus region of the human native ERα. Anti-ERβ primary antibodies were: rabbit polyclonal H-150 (Santa Cruz Biotechnology, Ca, USA) which recognizes an epitope mapping at the N-terminus region of human native ERβ and mouse monoclonal MCA1974 (Vector Laboratories, INC, Burlingame, CA) which recognizes an epitope mapping at the C-terminus region of human native ERβ. Rabbit polyclonal anti β-actin (Santa Cruz Biotechnology, Ca, USA) was also used as loading control. Fluorescein isothiocyanate (FITC) conjugated IgG (Sigma Aldrich, Milan, Italy), Texas-Red conjugated IgG (Vector Laboratories, INC, Burlingame, CA) and horseradish peroxidase conjugated IgG (Santa Cruz Biotechnology, Ca, USA) were used as secondary antibodies.

### Immunfluorescence assays

Following Percoll separation, sperm cells were rinsed three times with 0.5 mM Tris-HCl buffer, pH 7.5; then 10 μl of concentrated cell suspension was added to 250 μl drop of warm (37°C) TBS and allowed to settle onto slides in a humid chamber. The overlying solution was carefully pipetted off and replaced by absolute methanol for 7 minutes at -20°C. After methanol removal, sperm cells were washed in TBS, containing 0.1% Triton X-100 and were treated for immunofluorescence. Mouse monoclonal anti-AR (1:100), mouse monoclonal anti-P450arom (1:100), mouse monoclonal anti-ERα (1:50), rabbit polyclonal anti-ERβ (1:100) and mouse monoclonal anti-ERβ (1:50) have been utilized as primary antibodies with an overnight incubation at 4°C, while anti-mouse IgG Texas-red conjugated (1:100) and anti-rabbit IgG FITC conjugated (1:100) have been used as secondary antibodies with 1 hour incubation at room temperature. Sperm cells, incubated without the primary antibodies, were utilized as negative controls. Absorption controls were also performed by using the primary antibodies preabsorbed with an excess of the related purified antigens (aromatase purified protein, Hauptman-Woodward Medical Research Institute, Buffalo, NY, USA) (ERα blocking peptide: sc-8002P, ERβ 1 blocking peptide :sc-6820P, AR blocking peptide: sc-815P, Santa Cruz Biotechnology, Ca, USA) for 48 h at 4°C. The slides were immediately examined under an epifluorescence microscope (Olympus BX41) with a multiple fluorescence filter (U-DM-DA/FI/TX2) observing a minimum of 200 spermatozoa × slide (100× objective).

### Western blot analysis

After Percoll removal, sperm samples were resuspended in lysis buffer (62.5 mmol/L Tris-HCl, pH 6.8), 150 mM NaCl, 2% SDS, 1% Triton X100, 10% glycerol, 1 mM phenylmethylsulfonylfluoride, 0.2 mM Na_3_VO_4_, 1% aprotinin). Lysates were quantified using Bradford protein assay reagent [24]. Equal amounts of protein (20 μg) were boiled for 5 minutes, separated under denaturing conditions, by SDS-PAGE on 10% polyacrylamide Tris-glycine gels, and then electroblotted to nitrocellulose membrane. Non-specific sites were blocked with 5% non fat dry milk in 0.2% Tween-20 in Tris-buffered saline (TBS-T) for 1 hour at room temperature and the membrane was incubated overnight with anti- AR (1: 1500), anti- P450aromatase (1:1000), anti- ERα (1:500) and anti- ERβ (1:1000). Then antigen-antibody complexes were detected by incubation of the membranes with the appropriate secondary antibodies (anti-mouse or anti-rabbit horseradish peroxidase-conjugated, Amersham, USA) for 1 h at 22°C. The bound secondary antibodies were located with the ECL Plus Western blotting detection system (Amersham, USA) according to the manufacturer's instructions. Each membrane was exposed to the film for 2 minutes.

Protein extracts from MCF7 (breast cancer cell line) and LNCaP (prostate cancer cell line) were cultured as previously reported [9] and used as positive controls (see figure legends). Negative controls were prepared using sperm lysates where antigens were previously removed by pre-incubation with specific antibodies (1 h at room temperature) and subsequently immunoprecipitated with protein A/G -agarose.

Quantification of protein expression (related to β-actin) was performed using arbitrary densitometric units. Band intensities were compared by the one-way ANOVA.

## Results

### Immunofluorescence assays

**Figure 1:**
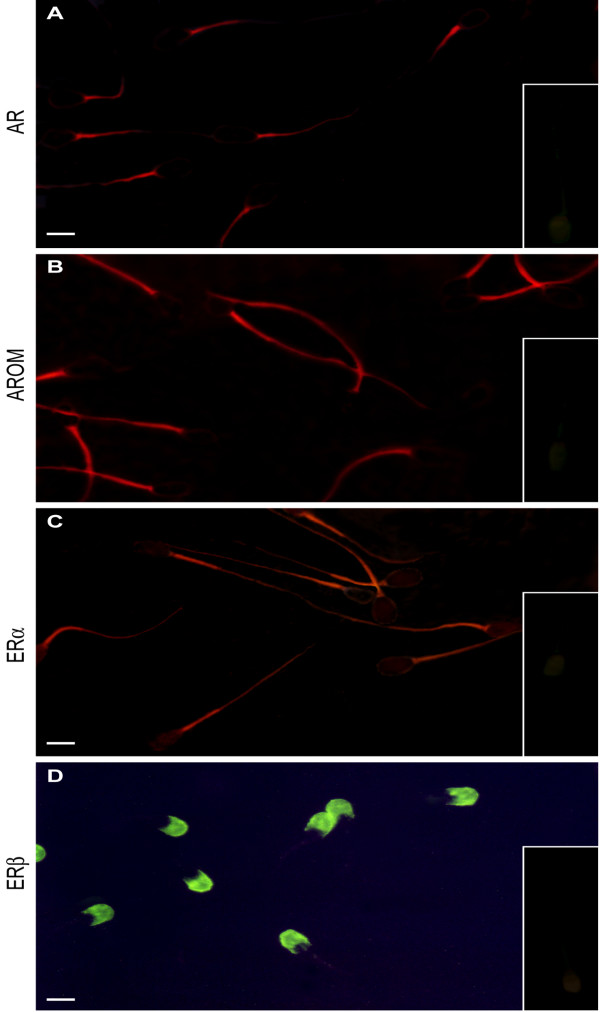
Immunofluorescence labelling of androgen and estrogen receptors in pig spermatozoa: A) AR red fluorescence in sperm proximal mid-piece. B) P450arom red brilliant light in the proximal tail of sperm with a diffuse labelling in the distal tail. C) ERα red fluorescence in the sperm mid-piece, together with a faint labelling in the tail. D) ERβ green intense light in the sperm acrosomal region. *Inserts*: immunonegative absorption controls. *Scale bars*: 5 μm.

A red fluorescence revealed AR in the proximal sperm mid-piece while the tail and the head were unstained (Figure 1A). P450arom has been detected in the tail of pig sperm with a red brilliant light in the proximal region and a diffuse labelling in the distal region (Figure 1B).

The two estrogen receptors revealed distinct cellular sites. A red fluorescence showed ERα in the mid-piece region of pig sperm, together with a faint labelling in the tail and no staining in the head (Figure 1C). Both the anti-ERβ antibodies localized ERβ only in the acrosomal region with a green intense light (Figure 1D) and a red fluorescence [see Additional file 1] respectively. Negative and absorption controls (inserts) were all immunonegative.

The immunofluorescence experiments were repeated 6 times with similar results.

### Immunoblotting

**Figure 2:**
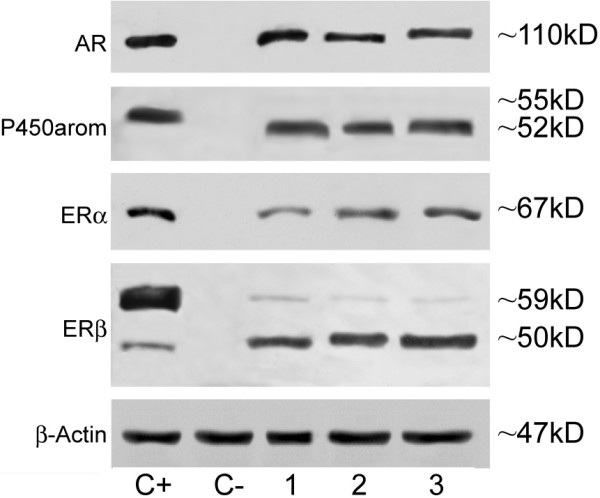
Western blotting analysis of AR, P450arom, ERα and ERβ of pig sperm extracts. Individual sperm samples: *lanes *1–3. LNCaP extracts were used as positive controls for AR and ERβ (*lanes *C+) while MCF7 extracts were used as positive controls for P450 arom and ERα (*lanes *C+). No bands in negative controls (*lanes *C-). β actin served as loading control. Molecular weights (KD) were indicated on the right of the blot.

A single band corresponding to the molecular weight values of ~110 kDa has been revealed in pig spermatozoa by the anti-AR antibody (Fig. 2, lanes 1–7) (103 ± 9.5 units). This band co-migrated with positive control band (LNCaP) (Fig 2, lanes C+) while no band has been observed in negative control (Fig 2, lane C-).

The anti- P450arom antibody detected in pig sperm extracts a single band at ~52 kDa (Fig 2, lanes 1–7) (132 ± 11 units). Positive control (MCF7) showed a ~55 kDa band (Fig 2: lane C+) while no band has been observed in negative control (Fig 2, lane C-).

The anti-ERα antibody revealed in pig sperm a single band corresponding to the molecular weight values of ~67 kDa (Fig 2, lanes 1–7) (78.7 ± 12.8 units) This band co-migrated with positive control band (MCF7) (Fig 2, lanes C+) while no band has been observed in negative control (Fig 2, lane C-). The anti- ERβ antibody showed two bands, at about ~59 kDa and at ~50 kDa (Fig 2, lanes 1–7). The ~59 kDa (32.4 ± 8 units) was weaker than the ~50 kDa (105 ± 11.6 units) (p < 0.001). Both the ERβ bands (Fig 2, lane C+) were observed in positive control (LNCaP) but not in negative control(Fig 2, lane C-).

## Discussion

The androgen and estrogen targeting of porcine male genital tract has been a subject of interest in different recent investigations. Androgen receptors have been detected in somatic cells of pig testes [25] and in epididymal cells of boar [26]. Furthermore, previous studies from our laboratory demonstrated in pig a differential ERα/ERβ expression in Leydig cells, spermatogonia and spermatocytes of young and adult testes [9] as well as the ERβ localization in epididymal epithelium [27]. Concordantly, in boar, distinct cellular ERα/ERβ expression patterns have been revealed in immature and mature male gonads [10] while both the ERs have been detected in epididymal cells [26]. In addition, cytochrome P450 has been demonstrated in Leydig cells of pig testes indicating them as possible sites of local estrogen production [28]. All these reports have suggested a role of both sex hormones, through a genomic action, in testicular and epididymal development as well as in sperm maturation.

However, to our knowledge, androgen and/or estrogen involvement in the biology of porcine mature sperm is scarcely known. The present study, for the first time, has provided evidence of AR, aromatase, ERα and ERβ expression in ejaculated sperm of pig, revealing their differential intra-cellular localization by immunofluorescence labeling. In fact, AR and ERα have been localised in the sperm mid-piece, aromatase has been detected in all the tail, while ERβ was confined in the acrosomal region of the male gamete. Western blot analysis has shown the AR and ERα full-length proteins, but it revealed also two ERβ species, at ~59 kDa and ~50 kDa, which could correspond to the long and short forms of ERβ recently reported in human sperm [20,29]. Furthermore, it has been also observed a slightly smaller aromatase band (52 Kda) showing a size similar to the pig testis aromatase [28].

Our results indicated that pig sperm share with human spermatozoa the expression of AR, P450arom, ERα and ERβ [19,20,30] but with some differences in the cellular sites.

We detected AR in the pig sperm mid-piece, agreeing to a previous report in human sperm [21]. In the latter paper, the AR co-localization with mitochondria led the authors to suggest a relation between AR and the cellular energy requirements. This hypothesis could be investigated also in pig spermatozoa.

Cytochrome P450aromatase, the enzyme catalysing the irreversible conversion of androgens in estrogens, has been localised in all the tail of pig sperm, indicating this region as possible site of estrogen biosynthesis. This finding is again consistent to previous data on human sperm, where a relation between the local estrogen production and sperm motility has been suggested [30,31]. Furthermore, decreased sperm motility has been also observed in two men with an inactivating mutation of the *CYP 19 *gene [32,33] and in aromatase-deficient mice [34].

Concerning estrogen receptors, we have detected ERα mainly in the proximal tail of pig sperm, such as in our previous work on human sperm where we had hypothesized the ERα involvement in cell survival and motility [19]. Impaired motility has been observed in sperm of mice lacking a functional ERα [35], even if the abnormal fluid reabsorption could be the main cause of damaged sperm motility in ERalpha Knockout mice [36]. Furthermore, we have to take in consideration that ERα has not still demonstrated in mouse testicular germ cell. Other authors reported altered motility in sperm of tamoxifene-treated rat, presumably through an ERα-mediated mechanism [22]. Therefore, it would be interesting to explore if the expression of P450arom and ERα in pig spermatozoa could be linked to estrogen regulation of sperm motility, as suggested in human and rodent male gametes. Conversely, we have found ERβ in the acrosomal cap of pig spermatozoa, differently from human sperm where, using the same antibody, we had detected ERβ in all the tail. This finding focuses a difference between the two species. Acrosomal cap is a cellular site closely related to the exocytotic event preceding the oocyte fertilization. Therefore, considering that estradiol is able to influence capacitation and acrosome reaction of human spermatozoa [37], the involvement of ERβ in pig fertilization process could be an interesting subject of future investigations.

The present work has detected in pig sperm AR and ERs showing molecular weights consistent with a genomic hormone action. However, fully differentiated gametes are known to have a densely packaged DNA, so a non classical action of these steroid hormones in pig spermatozoa could be also investigated. In fact, AR ad ERs have been reported to perform both transcriptional and transcription independent functions [38,39].

## Conclusion

This is the first report demonstrating that pig ejaculated spermatozoa express aromatase, estrogen and androgen receptors with a differential intra-cellular localization revealing a specie-specific expression pattern. Therefore, pig sperm could be considered as a potential estrogen source while the different hormone cellular sites suggest distinct roles of androgens and estrogens in pig sperm physiology. However, our findings represent a basic work and further studies need to elucidate the specific role of both sex hormones in the biology of mature pig sperm.

This work was supported by "Ministero dell'Università e della Ricerca Scientifica e Tecnologica" (Murst 60%).

## Authors' contributions

**RV: **the author responsible for performing the immunohistochemical experiments and participating in the analysis and interpretation of data.

**SA: **the author responsible for performing Western blot analysis.

**RP: **the author (chief of the Swine Artificial Insemination Centre) providing animals for sample collections.

**CA: **the author responsible for conception, design, analysis and interpretation of data as well as of drafting manuscript

## Supplementary Material

Additional File 1 ERbeta immunolocalization. A red fluorescence localized ERbeta in the sperm acrosomal region (Anti-ERβ primary antibody: mouse monoclonal MCA1974, Vector Laboratories, INC, Burlingame, CA) Click here for file
