# Supplementary figures and images for: Cytochrome P450arom, androgen and estrogen receptors in pig sperm
Source: Reprod Biol Endocrinol. 2007 Jun 6;5:23. doi: 10.1186/1477-7827-5-23 (PMC1894639; doi:10.1186/1477-7827-5-23)

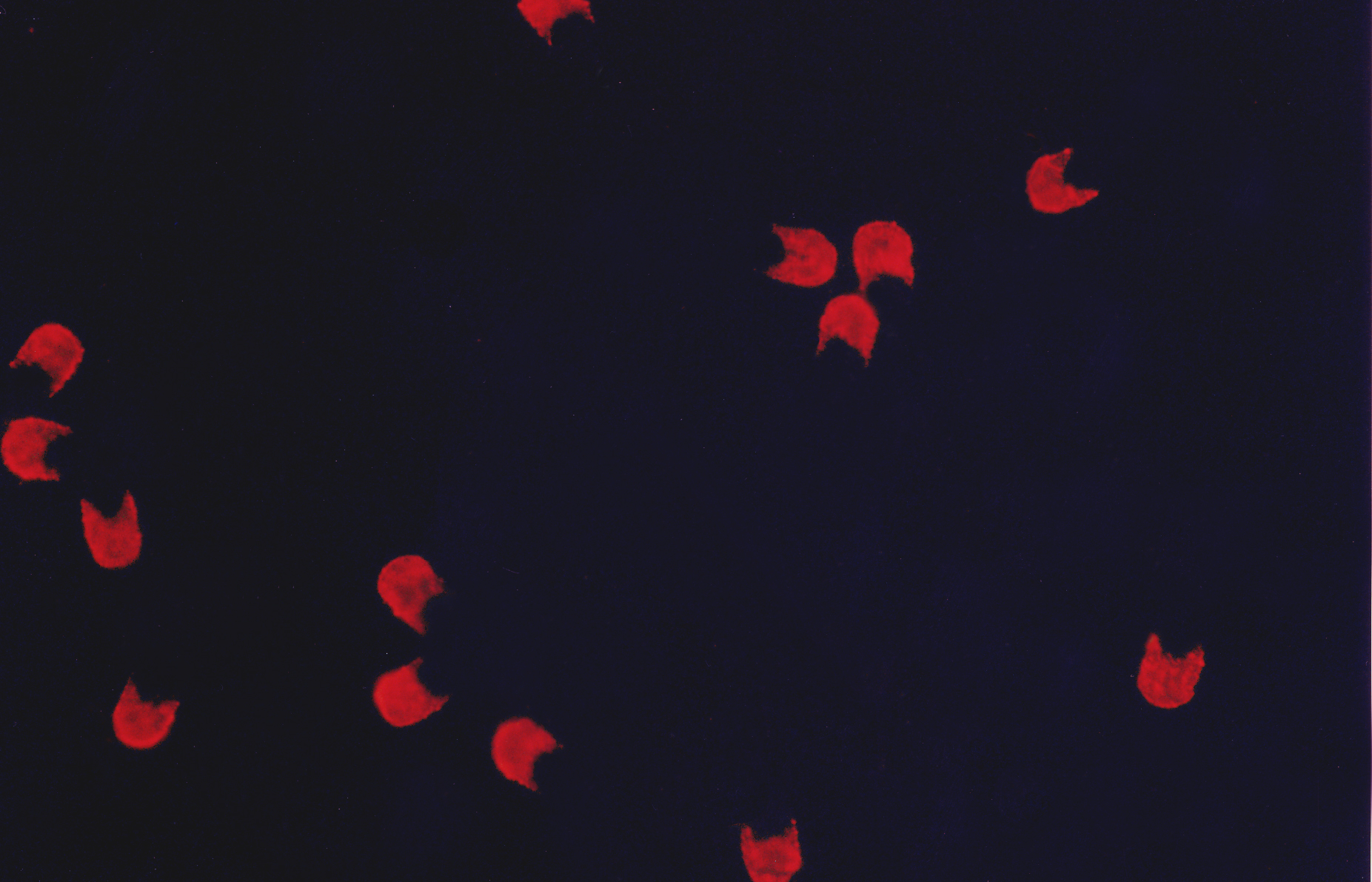

Supplement: Additional File 1 — ERbeta immunolocalization. A red fluorescence localized ERbeta in the sperm acrosomal region (Anti-ERβ primary antibody: mouse monoclonal MCA1974, Vector Laboratories, INC, Burlingame, CA) [file 1477-7827-5-23-S1.jpeg]
